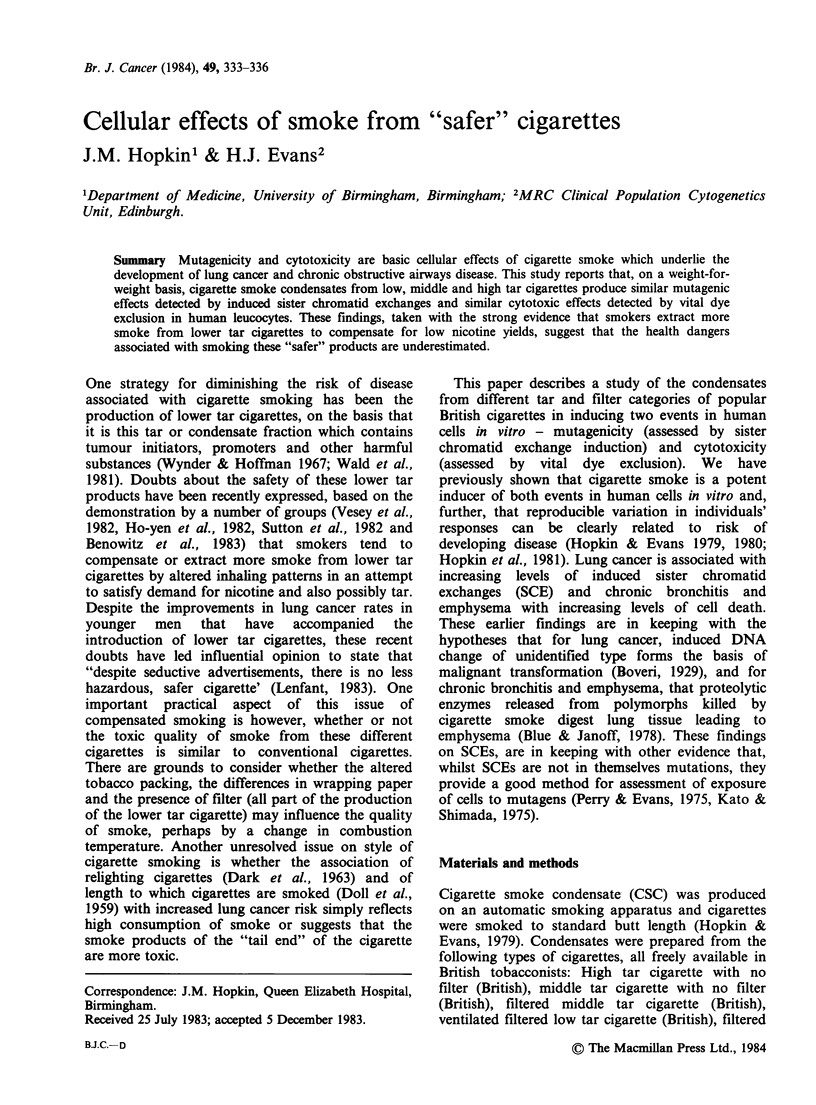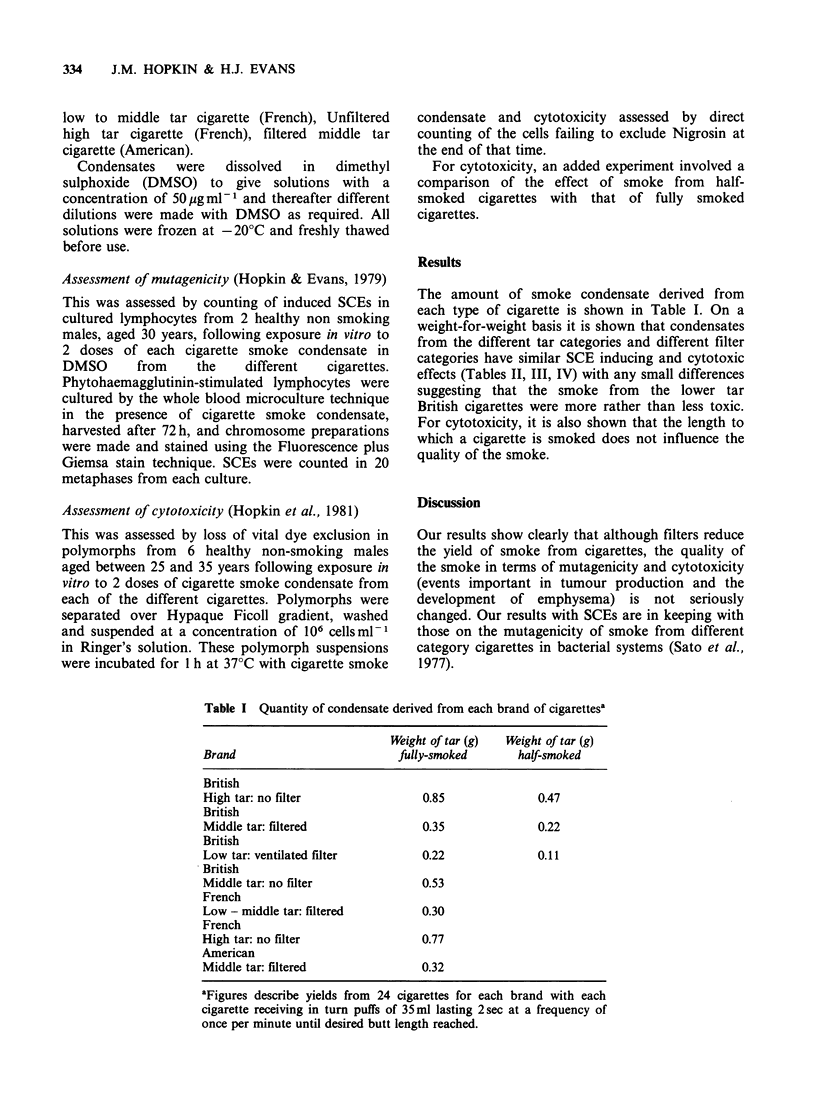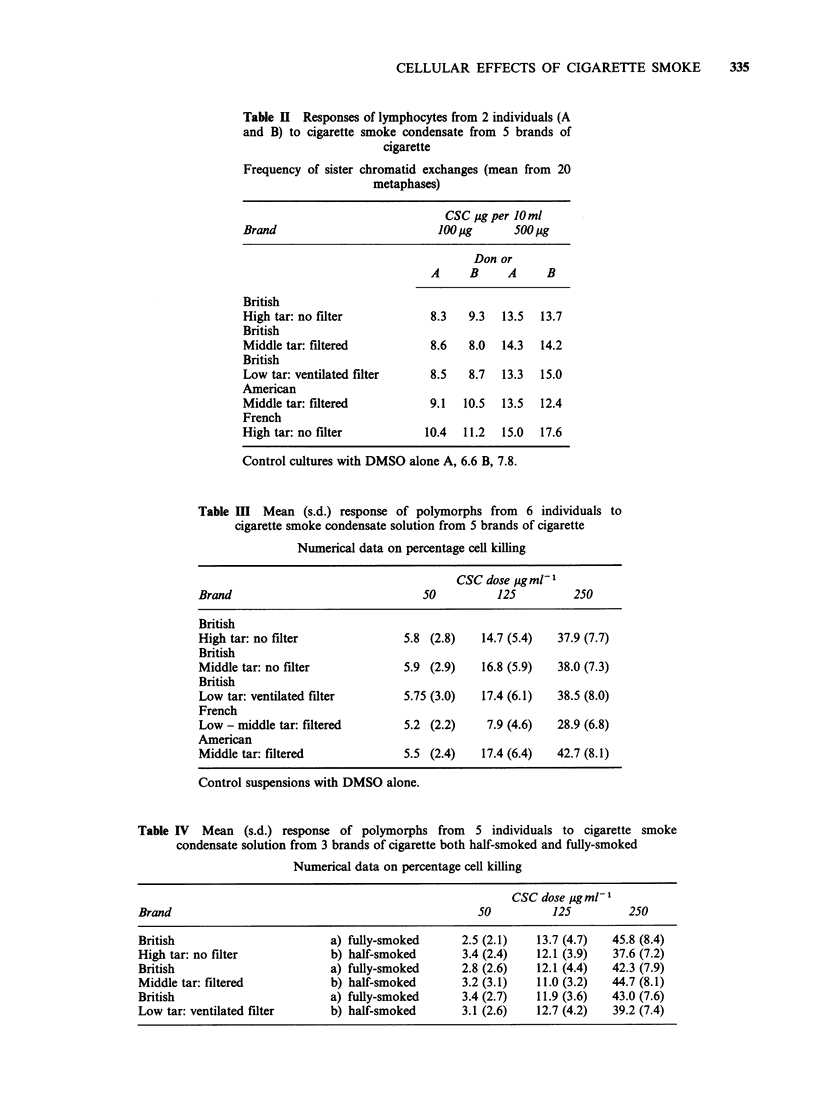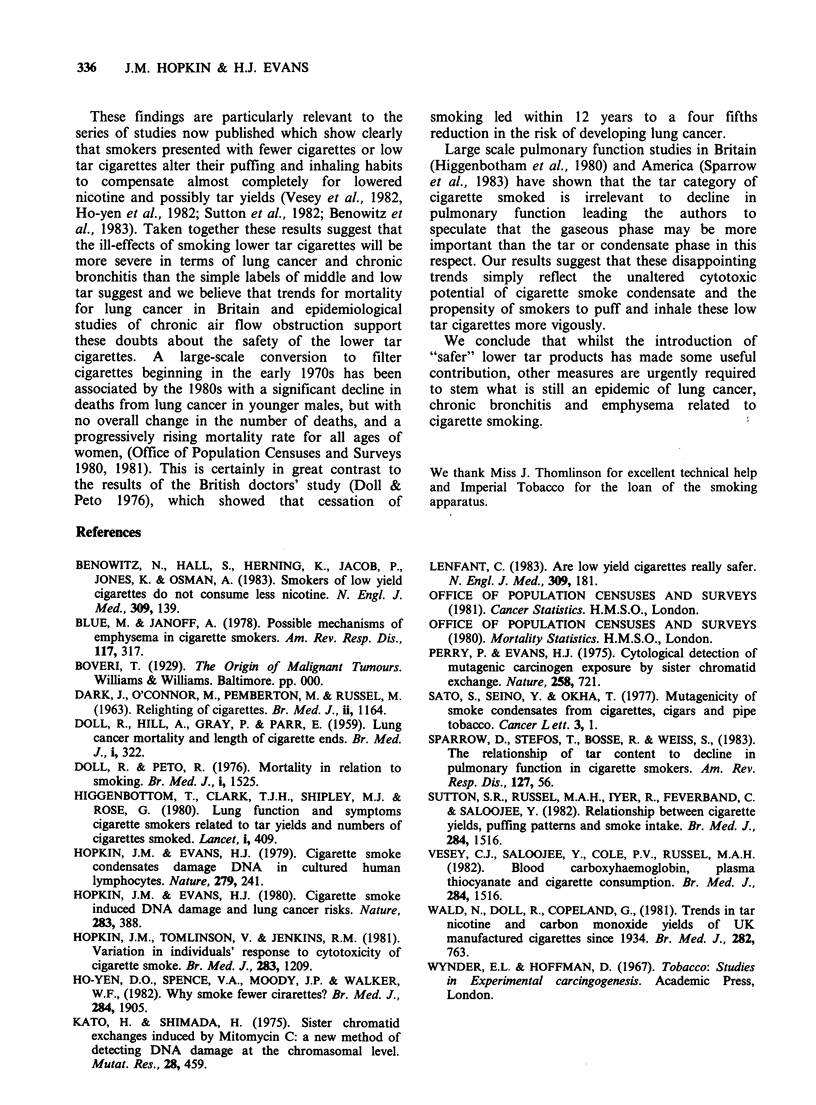# Cellular effects of smoke from "safer" cigarettes.

**DOI:** 10.1038/bjc.1984.52

**Published:** 1984-03

**Authors:** J. M. Hopkin, H. J. Evans

## Abstract

Mutagenicity and cytotoxicity are basic cellular effects of cigarette smoke which underlie the development of lung cancer and chronic obstructive airways disease. This study reports that, on a weight-for-weight basis, cigarette smoke condensates from low, middle and high tar cigarettes produce similar mutagenic effects detected by induced sister chromatid exchanges and similar cytotoxic effects detected by vital dye exclusion in human leucocytes. These findings, taken with the strong evidence that smokers extract more smoke from lower tar cigarettes to compensate for low nicotine yields, suggest that the health dangers associated with smoking these "safer" products are underestimated.


*Br. J. Cancer* (1984), **49**, 333–336

### Cellular effects of smoke from "safer" cigarettes

J.M. Hopkin[1] & H.J. Evans[2]

[1]*Department of Medicine, University of Birmingham, Birmingham;* [2]*MRC Clinical Population Cytogenetics Unit, Edinburgh.*

**Summary** Mutagenicity and cytotoxicity are basic cellular effects of cigarette smoke which underlie the development of lung cancer and chronic obstructive airways disease. This study reports that, on a weight-for-weight basis, cigarette smoke condensates from low, middle and high tar cigarettes produce similar mutagenic effects detected by induced sister chromatid exchanges and similar cytotoxic effects detected by vital dye exclusion in human leucocytes. These findings, taken with the strong evidence that smokers extract more smoke from lower tar cigarettes to compensate for low nicotine yields, suggest that the health dangers associated with smoking these "safer" products are underestimated.

One strategy for diminishing the risk of disease associated with cigarette smoking has been the production of lower tar cigarettes, on the basis that it is this tar or condensate fraction which contains tumour initiators, promoters and other harmful substances (Wynder & Hoffman 1967; Wald *et al.*, 1981). Doubts about the safety of these lower tar products have been recently expressed, based on the demonstration by a number of groups (Vesey *et al.*, 1982, Ho-yen *et al.*, 1982, Sutton *et al.*, 1982 and Benowitz *et al.*, 1983) that smokers tend to compensate or extract more smoke from lower tar cigarettes by altered inhaling patterns in an attempt to satisfy demand for nicotine and also possibly tar. Despite the improvements in lung cancer rates in younger men that have accompanied the introduction of lower tar cigarettes, these recent doubts have led influential opinion to state that "despite seductive advertisements, there is no less hazardous, safer cigarette' (Lenfant, 1983). One important practical aspect of this issue of compensated smoking is however, whether or not the toxic quality of smoke from these different cigarettes is similar to conventional cigarettes. There are grounds to consider whether the altered tobacco packing, the differences in wrapping paper and the presence of filter (all part of the production of the lower tar cigarette) may influence the quality of smoke, perhaps by a change in combustion temperature. Another unresolved issue on style of cigarette smoking is whether the association of relighting cigarettes (Dark *et al.*, 1963) and of length to which cigarettes are smoked (Doll *et al.*, 1959) with increased lung cancer risk simply reflects high consumption of smoke or suggests that the smoke products of the "tail end" of the cigarette are more toxic.

Correspondence: J.M. Hopkin, Queen Elizabeth Hospital, Birmingham.
Received 25 July 1983; accepted 5 December 1983.

This paper describes a study of the condensates from different tar and filter categories of popular British cigarettes in inducing two events in human cells *in vitro* – mutagenicity (assessed by sister chromatid exchange induction) and cytotoxicity (assessed by vital dye exclusion). We have previously shown that cigarette smoke is a potent inducer of both events in human cells *in vitro* and, further, that reproducible variation in individuals' responses can be clearly related to risk of developing disease (Hopkin & Evans 1979, 1980; Hopkin *et al.*, 1981). Lung cancer is associated with increasing levels of induced sister chromatid exchanges (SCE) and chronic bronchitis and emphysema with increasing levels of cell death. These earlier findings are in keeping with the hypotheses that for lung cancer, induced DNA change of unidentified type forms the basis of malignant transformation (Boveri, 1929), and for chronic bronchitis and emphysema, that proteolytic enzymes released from polymorphs killed by cigarette smoke digest lung tissue leading to emphysema (Blue & Janoff, 1978). These findings on SCEs, are in keeping with other evidence that, whilst SCEs are not in themselves mutations, they provide a good method for assessment of exposure of cells to mutagens (Perry & Evans, 1975, Kato & Shimada, 1975).

### Materials and methods

Cigarette smoke condensate (CSC) was produced on an automatic smoking apparatus and cigarettes were smoked to standard butt length (Hopkin & Evans, 1979). Condensates were prepared from the following types of cigarettes, all freely available in British tobacconists: High tar cigarette with no filter (British), middle tar cigarette with no filter (British), filtered middle tar cigarette (British), ventilated filtered low tar cigarette (British), filtered

© The Macmillan Press Ltd., 1984

low to middle tar cigarette (French), Unfiltered high tar cigarette (French), filtered middle tar cigarette (American).

Condensates were dissolved in dimethyl sulphoxide (DMSO) to give solutions with a concentration of $50\,\mu g\,ml^{-1}$ and thereafter different dilutions were made with DMSO as required. All solutions were frozen at −20°C and freshly thawed before use.

*Assessment of mutagenicity* (Hopkin & Evans, 1979)

This was assessed by counting of induced SCEs in cultured lymphocytes from 2 healthy non smoking males, aged 30 years, following exposure *in vitro* to 2 doses of each cigarette smoke condensate in DMSO from the different cigarettes. Phytohaemagglutinin-stimulated lymphocytes were cultured by the whole blood microculture technique in the presence of cigarette smoke condensate, harvested after 72 h, and chromosome preparations were made and stained using the Fluorescence plus Giemsa stain technique. SCEs were counted in 20 metaphases from each culture.

*Assessment of cytotoxicity* (Hopkin *et al.*, 1981)

This was assessed by loss of vital dye exclusion in polymorphs from 6 healthy non-smoking males aged between 25 and 35 years following exposure *in vitro* to 2 doses of cigarette smoke condensate from each of the different cigarettes. Polymorphs were separated over Hypaque Ficoll gradient, washed and suspended at a concentration of $10^6$ cells $ml^{-1}$ in Ringer's solution. These polymorph suspensions were incubated for 1 h at 37°C with cigarette smoke condensate and cytotoxicity assessed by direct counting of the cells failing to exclude Nigrosin at the end of that time.

For cytotoxicity, an added experiment involved a comparison of the effect of smoke from half-smoked cigarettes with that of fully smoked cigarettes.

### Results

The amount of smoke condensate derived from each type of cigarette is shown in Table I. On a weight-for-weight basis it is shown that condensates from the different tar categories and different filter categories have similar SCE inducing and cytotoxic effects (Tables II, III, IV) with any small differences suggesting that the smoke from the lower tar British cigarettes were more rather than less toxic. For cytotoxicity, it is also shown that the length to which a cigarette is smoked does not influence the quality of the smoke.

### Discussion

Our results show clearly that although filters reduce the yield of smoke from cigarettes, the quality of the smoke in terms of mutagenicity and cytotoxicity (events important in tumour production and the development of emphysema) is not seriously changed. Our results with SCEs are in keeping with those on the mutagenicity of smoke from different category cigarettes in bacterial systems (Sato *et al.*, 1977).

**Table I** Quantity of condensate derived from each brand of cigarettes[a]

| *Brand* | *Weight of tar (g) fully-smoked* | *Weight of tar (g) half-smoked* |
|---|---|---|
| British<br>High tar: no filter | 0.85 | 0.47 |
| British<br>Middle tar: filtered | 0.35 | 0.22 |
| British<br>Low tar: ventilated filter | 0.22 | 0.11 |
| British<br>Middle tar: no filter | 0.53 | |
| French<br>Low – middle tar: filtered | 0.30 | |
| French<br>High tar: no filter | 0.77 | |
| American<br>Middle tar: filtered | 0.32 | |

[a]Figures describe yields from 24 cigarettes for each brand with each cigarette receiving in turn puffs of 35 ml lasting 2 sec at a frequency of once per minute until desired butt length reached.

**Table II** Responses of lymphocytes from 2 individuals (A and B) to cigarette smoke condensate from 5 brands of cigarette

Frequency of sister chromatid exchanges (mean from 20 metaphases)

| *Brand* | *CSC µg per 10 ml* *100 µg* | | *500 µg* | |
|---|---|---|---|---|
| | *Don or* *A* | *B* | *A* | *B* |
| British High tar: no filter | 8.3 | 9.3 | 13.5 | 13.7 |
| British Middle tar: filtered | 8.6 | 8.0 | 14.3 | 14.2 |
| British Low tar: ventilated filter | 8.5 | 8.7 | 13.3 | 15.0 |
| American Middle tar: filtered | 9.1 | 10.5 | 13.5 | 12.4 |
| French High tar: no filter | 10.4 | 11.2 | 15.0 | 17.6 |

Control cultures with DMSO alone A, 6.6 B, 7.8.

**Table III** Mean (s.d.) response of polymorphs from 6 individuals to cigarette smoke condensate solution from 5 brands of cigarette

Numerical data on percentage cell killing

| *Brand* | *CSC dose µg ml⁻¹* *50* | *125* | *250* |
|---|---|---|---|
| British High tar: no filter | 5.8 (2.8) | 14.7 (5.4) | 37.9 (7.7) |
| British Middle tar: no filter | 5.9 (2.9) | 16.8 (5.9) | 38.0 (7.3) |
| British Low tar: ventilated filter | 5.75 (3.0) | 17.4 (6.1) | 38.5 (8.0) |
| French Low – middle tar: filtered | 5.2 (2.2) | 7.9 (4.6) | 28.9 (6.8) |
| American Middle tar: filtered | 5.5 (2.4) | 17.4 (6.4) | 42.7 (8.1) |

Control suspensions with DMSO alone.

**Table IV** Mean (s.d.) response of polymorphs from 5 individuals to cigarette smoke condensate solution from 3 brands of cigarette both half-smoked and fully-smoked

Numerical data on percentage cell killing

| *Brand* | | *CSC dose µg ml⁻¹* *50* | *125* | *250* |
|---|---|---|---|---|
| British | a) fully-smoked | 2.5 (2.1) | 13.7 (4.7) | 45.8 (8.4) |
| High tar: no filter | b) half-smoked | 3.4 (2.4) | 12.1 (3.9) | 37.6 (7.2) |
| British | a) fully-smoked | 2.8 (2.6) | 12.1 (4.4) | 42.3 (7.9) |
| Middle tar: filtered | b) half-smoked | 3.2 (3.1) | 11.0 (3.2) | 44.7 (8.1) |
| British | a) fully-smoked | 3.4 (2.7) | 11.9 (3.6) | 43.0 (7.6) |
| Low tar: ventilated filter | b) half-smoked | 3.1 (2.6) | 12.7 (4.2) | 39.2 (7.4) |

These findings are particularly relevant to the series of studies now published which show clearly that smokers presented with fewer cigarettes or low tar cigarettes alter their puffing and inhaling habits to compensate almost completely for lowered nicotine and possibly tar yields (Vesey *et al.*, 1982, Ho-yen *et al.*, 1982; Sutton *et al.*, 1982; Benowitz *et al.*, 1983). Taken together these results suggest that the ill-effects of smoking lower tar cigarettes will be more severe in terms of lung cancer and chronic bronchitis than the simple labels of middle and low tar suggest and we believe that trends for mortality for lung cancer in Britain and epidemiological studies of chronic air flow obstruction support these doubts about the safety of the lower tar cigarettes. A large-scale conversion to filter cigarettes beginning in the early 1970s has been associated by the 1980s with a significant decline in deaths from lung cancer in younger males, but with no overall change in the number of deaths, and a progressively rising mortality rate for all ages of women, (Office of Population Censuses and Surveys 1980, 1981). This is certainly in great contrast to the results of the British doctors' study (Doll & Peto 1976), which showed that cessation of smoking led within 12 years to a four fifths reduction in the risk of developing lung cancer.

Large scale pulmonary function studies in Britain (Higgenbotham *et al.*, 1980) and America (Sparrow *et al.*, 1983) have shown that the tar category of cigarette smoked is irrelevant to decline in pulmonary function leading the authors to speculate that the gaseous phase may be more important than the tar or condensate phase in this respect. Our results suggest that these disappointing trends simply reflect the unaltered cytotoxic potential of cigarette smoke condensate and the propensity of smokers to puff and inhale these low tar cigarettes more vigously.

We conclude that whilst the introduction of "safer" lower tar products has made some useful contribution, other measures are urgently required to stem what is still an epidemic of lung cancer, chronic bronchitis and emphysema related to cigarette smoking.

We thank Miss J. Thomlinson for excellent technical help and Imperial Tobacco for the loan of the smoking apparatus.

### References

BENOWITZ, N., HALL, S., HERNING, K., JACOB, P., JONES, K. & OSMAN, A. (1983). Smokers of low yield cigarettes do not consume less nicotine. *N. Engl. J. Med.*, **309**, 139.

BLUE, M. & JANOFF, A. (1978). Possible mechanisms of emphysema in cigarette smokers. *Am. Rev. Resp. Dis.*, **117**, 317.

BOVERI, T. (1929). *The Origin of Malignant Tumours.* Williams & Williams. Baltimore. pp. 000.

DARK, J., O'CONNOR, M., PEMBERTON, M. & RUSSEL, M. (1963). Relighting of cigarettes. *Br. Med. J.*, **ii**, 1164.

DOLL, R., HILL, A., GRAY, P. & PARR, E. (1959). Lung cancer mortality and length of cigarette ends. *Br. Med. J.*, **i**, 322.

DOLL, R. & PETO, R. (1976). Mortality in relation to smoking. *Br. Med. J.*, **i**, 1525.

HIGGENBOTTOM, T., CLARK, T.J.H., SHIPLEY, M.J. & ROSE, G. (1980). Lung function and symptoms cigarette smokers related to tar yields and numbers of cigarettes smoked. *Lancet*, **i**, 409.

HOPKIN, J.M. & EVANS, H.J. (1979). Cigarette smoke condensates damage DNA in cultured human lymphocytes. *Nature*, **279**, 241.

HOPKIN, J.M. & EVANS, H.J. (1980). Cigarette smoke induced DNA damage and lung cancer risks. *Nature*, **283**, 388.

HOPKIN, J.M., TOMLINSON, V. & JENKINS, R.M. (1981). Variation in individuals' response to cytotoxicity of cigarette smoke. *Br. Med. J.*, **283**, 1209.

HO-YEN, D.O., SPENCE, V.A., MOODY, J.P. & WALKER, W.F., (1982). Why smoke fewer cirarettes? *Br. Med. J.*, **284**, 1905.

KATO, H. & SHIMADA, H. (1975). Sister chromatid exchanges induced by Mitomycin C: a new method of detecting DNA damage at the chromasomal level. *Mutat. Res.*, **28**, 459.

LENFANT, C. (1983). Are low yield cigarettes really safer. *N. Engl. J. Med.*, **309**, 181.

OFFICE OF POPULATION CENSUSES AND SURVEYS (1981). *Cancer Statistics.* H.M.S.O., London.

OFFICE OF POPULATION CENSUSES AND SURVEYS (1980). *Mortality Statistics.* H.M.S.O., London.

PERRY, P. & EVANS, H.J. (1975). Cytological detection of mutagenic carcinogen exposure by sister chromatid exchange. *Nature*, **258**, 721.

SATO, S., SEINO, Y. & OKHA, T. (1977). Mutagenicity of smoke condensates from cigarettes, cigars and pipe tobacco. *Cancer Lett.* **3**, 1.

SPARROW, D., STEFOS, T., BOSSE, R. & WEISS, S., (1983). The relationship of tar content to decline in pulmonary function in cigarette smokers. *Am. Rev. Resp. Dis.*, **127**, 56.

SUTTON, S.R., RUSSEL, M.A.H., IYER, R., FEVERBAND, C. & SALOOJEE, Y. (1982). Relationship between cigarette yields, puffing patterns and smoke intake. *Br. Med. J.*, **284**, 1516.

VESEY, C.J., SALOOJEE, Y., COLE, P.V., RUSSEL, M.A.H. (1982). Blood carboxyhaemoglobin, plasma thiocyanate and cigarette consumption. *Br. Med. J.*, **284**, 1516.

WALD, N., DOLL, R., COPELAND, G., (1981). Trends in tar nicotine and carbon monoxide yields of UK manufactured cigarettes since 1934. *Br. Med. J.*, **282**, 763.

WYNDER, E.L. & HOFFMAN, D. (1967). *Tobacco: Studies in Experimental carcingogenesis.* Academic Press, London.